# Dental calculus detection using the VistaCam

**DOI:** 10.1002/cre2.42

**Published:** 2016-09-13

**Authors:** Fardad Shakibaie, Laurence J. Walsh

**Affiliations:** ^1^ School of Dentistry The University of Queensland Brisbane Queensland Australia

**Keywords:** dental calculus, detection, fluorescence imaging, VistaCam

## Abstract

The VistaCam® intra‐oral camera system (Dürr Dental, Bietigheim‐Bissingen, Germany) is a fluorescence system using light emitting diodes that produce a 405‐nm violet light. This wavelength has potential application for detection of dental calculus based on red emissions from porphyrin molecules. This study assessed the digital scores obtained for both supragingival and subgingival calculus on 60 extracted teeth and compared these with lesions of dental caries. It has also examined the effect of saliva and blood on the fluorescence readings for dental calculus. VistaCam fluorescence scores for both supragingival (1.7–3.3) and subgingival calculus (1.3–2.4) were higher than those for sound root surfaces (0.9–1.1) and dental caries (0.9–2.2) (*p* < .05). The readings for calculus samples were not affected by the presence of saliva or blood. These results suggest that the use of violet light fluorescence could be a possible adjunct to clinical examination for deposits of dental calculus.

Deposits of supragingival and subgingival calculus contain a range of periodontopathogenic microorganisms and their products, such that removal of these is an essential part of the treatment of periodontitis (Bird, Shakibaie, Gemmell, Polak, & Seymour, 2001; Shakibaie, Gemmell, & Bird, 2001). As supragingival calculus may have a similar color to teeth, thin deposits of this may be overlooked during clinical examination. A range of methods have been suggested to assist in the detection of calculus deposits, including fluorescence imaging and differential reflectometry (Shakibaie & Walsh, 2012; Shakibaie & Walsh, 2014; Shakibaie & Walsh, 2015c; Walsh & Shakibaie, 2007; Shakibaie & Walsh, 2016). These same methods can be used to help determine the endpoint of successful debridement (Shakibaie, George, & Walsh, 2011; Shakibaie & Walsh, 2015a).

The fluorescence phenomenon of violet light eliciting visible red light emissions from calculus deposits has been described in the literature (Buchalla, Lennon, & Attin, 2004). Given the availability of violet light‐equipped dental imaging systems, it is of interest to explore the potential application of this concept in clinical practice. The VistaCam system (Dürr Dental, Bietigheim‐Bissingen, Germany) emits violet light at a 405‐nm wavelength and has been used for enhanced detection of dental caries and mature deposits of dental plaque (Eberhart, Frentzen, & Thoms, 2007; Tomczyk, Komarnitki, Zalewska, Lekszycki, & Olczak‐Kowalczyk, 2014). The violet excitation light is removed by a filter located in front of the sensor. The fluorescence signals are converted to numbers using a look‐up table and shown as a pseudo‐color image (Jablonski‐Momeni, Liebegall, Stoll, Heinzel‐Gutenbrunner, & Pieper, 2013).

As the primary use of the VistaCam has been for the assessment of dental caries, there are as yet no data on dental calculus that could be used to distinguish deposits of dental calculus from healthy tooth and root surfaces. In recent studies, we have determined the VistaCam digital readings for healthy tooth surfaces (such as enamel and dentine root surfaces), which range from 0.9 to 1.1, and those for carious lesions, which range from 0.9 to 2.2 (Shakibaie & Walsh, 2015b). The present study was undertaken to establish the range of scores for dental calculus and also to investigate the influence of contaminating saliva and blood on these scores

## 2 MATERIALS AND METHODS

### 2.1 Experimental design

Sixty extracted human permanent teeth were collected with the approval of the institutional ethics committee (Reference no: 2003000040) from a dental school exodontia clinic. All teeth were from adults aged 18 years or more. The teeth were examined under 20× magnifications to exclude those with defects, then cleaned to remove external stains from the enamel. The teeth were stored in distilled water to maintain their hydration. A total of 20 teeth per group were selected for the supragingival calculus group, the subgingival calculus group, and the caries group.

**Figure 1:**
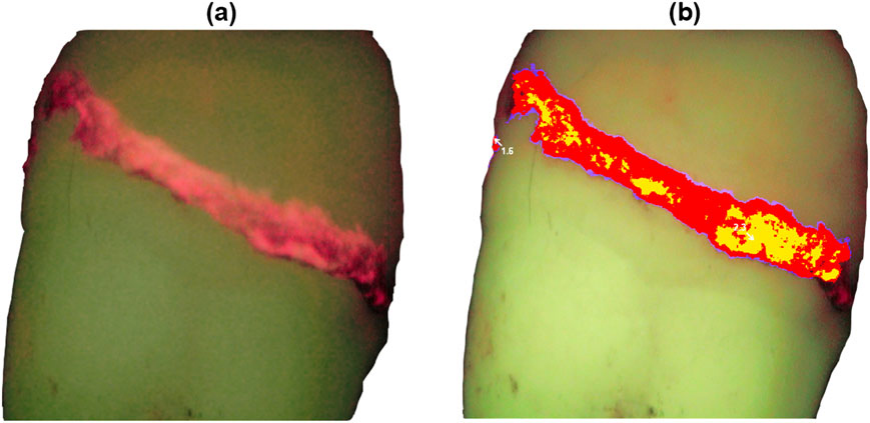
(a) Fluorescence image of supragingival calculus, viewed using the VistaCam, showing typical red emissions. (b) The corresponding pseudo reference image

The VistaCam intra‐oral camera was connected via USB to a laptop computer. The manufacturer's supplied spacer was applied to the handpiece to give a consistent distance from the tooth surface and constant 90° angulation. Imaging was done in a dark room to avoid the influence of external room lighting. The teeth were examined first in the moist state (free of gross surface water). The fluorescence image was analyzed using the supplied Dürr DBSWIN software, to generate a pseudo reference image, which provided the maximum scores (Figure 1).

The effect of saliva and blood on the readings was then examined by applying first stimulated saliva and then anti‐coagulated venous blood. Both were collected from a single healthy male volunteer with approval from the institutional ethics committee (Reference no: 2006000701). The surface of the tooth to be imaged was covered with 40 μL of stimulated saliva. After imaging, this was rinsed away and then replaced with a mixture of heparinized blood (diluted 1:8 in distilled water) prior to undertaking further imaging. This treatment was intended to replicate the effect of gingival bleeding, which could occur under clinical conditions, giving contamination of the tooth surfaces by saliva mixed with small amounts of blood.

### 2.2 Data analysis

The VistaCam numerical scores for fluorescence were analyzed using GraphPad Prism version 6 software, to compare the effects of sample type (analysis of variance [ANOVA]), and surface conditions (moist, saliva coated, and blood coated; repeated measures ANOVA). Data sets were examined for normality. The sample size chosen gave a power of almost 100% at *α* = 0.05 setting.

## 3 RESULTS

**Figure 2:**
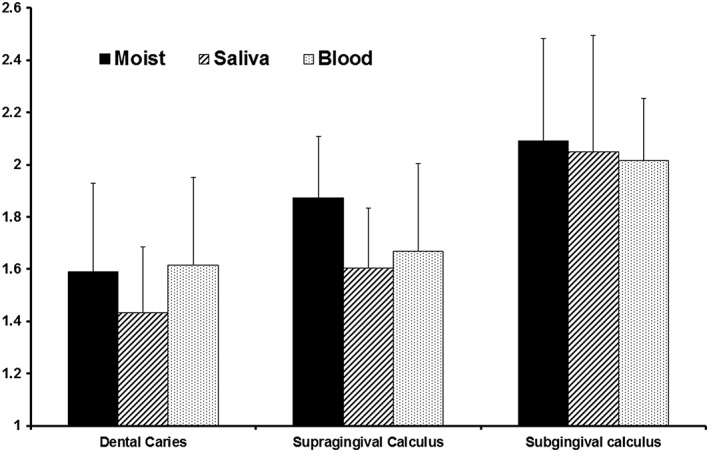
VistaCam fluorescence data showing means and standard deviations for various tooth samples. The Y axis is the fluorescence scores, in arbitrary fluorescence units

Mean VistaCam scores from samples are shown in Figure 2, while Table 1 presents summary results for numerical ranges. The state of the surface did not significantly affect the fluorescence scores, because there were no statistically significant differences between moist, saliva‐, and blood‐coated surfaces for each sample type (*p* > .05).

**Table 1:** The range of scores for samples under different conditions

Sample	Dry	Saliva	Blood
Supragingival calculus	1.7–3.3	1.3–3	1.7–2.4
Subgingival calculus	1.3–2.4	1.2–2.1	1.2–2.3
Dental caries	0.9–2.2	0.9–1.8	0.9–2.2

For moist samples, fluorescence scores for both supragingival calculus and subgingival calculus were similar (*p* = .1267), and both were significantly higher (*p* < .05) than those for dental caries (0.9–2.2). The same trend was seen for sample surfaces when covered with saliva or blood.

## 4 DISCUSSION

This study shows that fluorescence scores for the VistaCam are greater for calculus deposits than for dental caries. This finding suggests that the applications of the fluorescence scores from this device should be extended to include the assessment of dental calculus. This could be particularly useful for thin deposits of supragingival calculus spread over tooth surfaces, which may be similar in color to the underlying enamel, such as those located on lingual surfaces of mandibular incisor teeth. The present study also found that contaminating saliva and blood on dental calculus did not greatly affect the fluorescence readings. This point is relevant clinically because one would expect to encounter saliva or bleeding in areas where calculus is present.

The VistaCam system in its present form has not been designed to image root surfaces within narrow periodontal pockets to show deposits of subgingival calculus. To achieve this would require the use of solid fiber optics to deliver the 405‐nm excitation wavelength of light and collect the red fluorescence emissions. Use of a periscope, side‐firing, or lateral emission design on the optical element within the pocket would be necessary.

Scores from the VistaCam are high for both dental calculus and dental caries as both fluoresce are red under violet or ultraviolet light illumination (Thoms, 2006; Tassery et al., 2013). When considered against the narrow range for healthy tooth structure (0.9–1.1), the higher numerical scores for dental calculus (1.3–2.4) could aid differentiation between calculus and healthy roots (Shakibaie & Walsh, 2015b). This could assist in defining endpoints for treatment when small deposits of supragingival calculus remain after periodontal debridement and have been overlooked. Typically, visual clinical examination is used to identify deposits of supragingival calculus, but this is imperfect when clinicians fail to isolate and dry the teeth very well when checking the debrided surfaces.

The situation is more complex for subgingival calculus versus root surface caries as there is considerable overlap between the ranges for typical VistaCam scores for calculus (1.3–2.4) and those for dental caries (0.9–2.2). Clinically, the topography of the tooth surface usually differs between subgingival calculus and subgingival caries, so that information would need to be taken into account, rather than relying on a numerical score alone to inform the diagnosis.

This study is important from a clinical point of view because it suggests that, as a diagnostic device, the VistaCam could have dual clinical utility for identifying deposits of calculus as well as dental caries. The elevated values for supragingival calculus make the approach useful to guide the endpoint of supragingival calculus removal. This aligns with past work showing that the use of auxiliary diagnostic devices can support the clinician to provide endpoints for care. The challenge with the VistaCam device in its current optical configuration is that it is not designed for use in the periodontal pocket environment, but if suitable changes were made in this direction, the results from this study show promise for its eventual use for guiding clinicians in the detection of subgingival calculus. The overall approach then would be similar to using laser fluorescence to inform the decision around stopping or continuing subgingival calculus debridement (Folwaczny, Heym, Mehl, & Hickel, 2002; Krause, Braun, & Frentzen, 2003; Folwaczny, Heym, Mehl, & Hickel, 2004; Shakibaie & Walsh, 2012).

## 5 CONCLUSION

This study provides direct laboratory evidence for the performance of the VistaCam device for the detection of calculus. The fluorescence numerical readings from the VistaCam are high for both supragingival and subgingival calculus, regardless of whether the surface was moist or was coated with saliva or blood.

### 5.1 Clinical relevance

#### 5.1.1 Scientific rationale for the study

Bacterial products such as porphyrins present in dental calculus give visible red fluorescence emissions under violet light excitation. Thus, the VistaCam system should show high fluorescence readings when such deposits are encountered on the surfaces of teeth.

#### 5.1.2 Principal findings

The fluorescence scores for dental calculus, whether supragingival or subgingival, are high, and greater than typical scores for dental caries.

#### 5.1.3 Practical implications

When using the VistaCam as an adjunct to conventional mirror and probe clinical examinations, clinicians need to be aware that red fluorescence on a root surface can be due to the presence of dental calculus as well as dental caries. Values are not affected by the moist state of the surface being measured, or by overlying saliva or blood.

## CONFLICTS OF INTEREST

The authors declare that they have no conflict of interests. The study was not funded by the manufacturer of the VistaCam system used in the study.
